# Trust in and Acceptance of Artificial Intelligence Applications in Medicine: Mixed Methods Study

**DOI:** 10.2196/47031

**Published:** 2024-01-17

**Authors:** Daria Shevtsova, Anam Ahmed, Iris W A Boot, Carmen Sanges, Michael Hudecek, John J L Jacobs, Simon Hort, Hubertus J M Vrijhoef

**Affiliations:** 1 Panaxea bv Den Bosch Netherlands; 2 Vrije Universiteit Amsterdam Amsterdam Netherlands; 3 Universitätsklinikum Würzburg Würzburg Germany; 4 Ortec bv Zoetermeer Netherlands; 5 Fraunhofer Institute for Production Technology Aachen Germany

**Keywords:** trust, acceptance, artificial intelligence, medicine, mixed methods, rapid review, survey

## Abstract

**Background:**

Artificial intelligence (AI)–powered technologies are being increasingly used in almost all fields, including medicine. However, to successfully implement medical AI applications, ensuring trust and acceptance toward such technologies is crucial for their successful spread and timely adoption worldwide. Although AI applications in medicine provide advantages to the current health care system, there are also various associated challenges regarding, for instance, data privacy, accountability, and equity and fairness, which could hinder medical AI application implementation.

**Objective:**

The aim of this study was to identify factors related to trust in and acceptance of novel AI-powered medical technologies and to assess the relevance of those factors among relevant stakeholders.

**Methods:**

This study used a mixed methods design. First, a rapid review of the existing literature was conducted, aiming to identify various factors related to trust in and acceptance of novel AI applications in medicine. Next, an electronic survey including the rapid review–derived factors was disseminated among key stakeholder groups. Participants (N=22) were asked to assess on a 5-point Likert scale (1=irrelevant to 5=relevant) to what extent they thought the various factors (N=19) were relevant to trust in and acceptance of novel AI applications in medicine.

**Results:**

The rapid review (N=32 papers) yielded 110 factors related to trust and 77 factors related to acceptance toward AI technology in medicine. Closely related factors were assigned to 1 of the 19 overarching umbrella factors, which were further grouped into 4 categories: human-related (ie, the type of institution AI professionals originate from), technology-related (ie, the explainability and transparency of AI application processes and outcomes), ethical and legal (ie, data use transparency), and additional factors (ie, AI applications being environment friendly). The categorized 19 umbrella factors were presented as survey statements, which were evaluated by relevant stakeholders. Survey participants (N=22) represented researchers (n=18, 82%), technology providers (n=5, 23%), hospital staff (n=3, 14%), and policy makers (n=3, 14%). Of the 19 factors, 16 (84%) human-related, technology-related, ethical and legal, and additional factors were considered to be of high relevance to trust in and acceptance of novel AI applications in medicine. The patient’s gender, age, and education level were found to be of low relevance (3/19, 16%).

**Conclusions:**

The results of this study could help the implementers of medical AI applications to understand what drives trust and acceptance toward AI-powered technologies among key stakeholders in medicine. Consequently, this would allow the implementers to identify strategies that facilitate trust in and acceptance of medical AI applications among key stakeholders and potential users.

## Introduction

### Artificial Intelligence

Artificial intelligence (AI) is commonly defined as a computer system that uses statistical models, diverse algorithms, and self-modifying systems to make predictions and decisions based on its own aggregated experience. It can therefore perform tasks that usually require or even surpass the human level of intelligence [1,2]. AI has been increasingly integrated in the health care sector, where it helps with administrative workflows, diagnostic image analysis, robotic surgery, and clinical decision-making. Consequently, medical AI applications allow, amongst other things, earlier disease detection, patient-tailored treatments, and more efficient follow-ups, which should drive the health care costs down upon implementation [3]. Although medical AI applications provide various advantages to the current health care system, such as increased efficiency and improved workflows [4], there are also various challenges associated with AI implementation. For instance, a large share of potential users has concerns over privacy issues [5,6]. Equity and fairness are other important concerns, since there is a risk of perpetuating bias within data sets being adopted by AI technology [5,7,8]. Further, the implementation of AI into medical practice raises the question of accountability, since it is currently unclear whether technology developers, hospitals, or regulators should be responsible for mistakes or undesirable outcomes from the use of an AI application.

### Trust and Acceptance

To ensure successful implementation of medical AI applications, it is essential to build trust in and acceptance of AI technology among its users [9,10]. In this study, a model of key drivers of trust in and acceptance of AI systems was used [11]. According to this model, trust is influenced by 4 drivers: current safeguards, job impact of AI, familiarity of AI, and AI uncertainty. Current safeguards indicate the belief that current regulations and laws are adequate for ensuring the safety of AI and the protection of people who use it. Job impact refers to the belief that there will be more jobs generated than eliminated due to AI implementation. Familiarity with AI is the level of understanding of how AI technology works and how AI applications are used. These 3 drivers have a positive influence on trust, with current safeguards being its strongest driver. The fourth driver, AI uncertainty, impacts trust in a negative way. It implies the belief that the impact of AI on society is unpredictable and the technology is still not fully explored. Overall, these drivers influence the extent to which people trust the AI system and believe it to be trustworthy. Trust, then, is a large contributor to the level of acceptance, which is the extent to which people accept or approve of AI and are willing to use it without resistance [11]. In the scientific literature, trust can be defined in different ways. In this study, we used literature-derived definitions of trust and acceptance in the context of AI implementation, namely:

- Trust is the belief of an individual that an AI application will do what it promises [12,13].
- Acceptance is the willingness of an individual to use the AI application in medicine [14].

Therefore, it can be argued that acceptance of an AI application depends on trust people have toward this technology [11,15,16]. At the same time, people can often accept their usage of technologies without necessarily trusting them [17]. Therefore, it is important to consider the 2 concepts separately as well as together.

Overall, widespread trust in and acceptance of an AI application is crucial for successful introduction and implementation of the technology. Failure to ensure trust in and acceptance of AI technology would pose the risk of “stifling innovation” and causing unnecessary “opportunity costs” [18]. The lack of trust in AI applications in medicine impedes their adoption in health care, compounded by inadequate public assurance and attention to concerns, thereby exacerbating these challenges. In addition, the anticipated benefits of AI-based innovations can coexist with significant acceptance barriers [15,18-21].

Investigating what factors contribute to trust in and acceptance of AI technology in medicine would help us understand how to make the implementation and regulatory approval of AI-powered advanced therapy manufacturing systems as efficient as possible. This can be achieved by collecting insights into stakeholders’ perspectives with regard to trust and acceptance toward medical AI applications [2,22]. Factors contributing to trust and acceptance toward medical AI applications can be attributed a different weight by various groups of stakeholders with distinct roles in AI.

### Study Objectives

Since AI applications are still relatively new, users and providers are hesitant to trust and accept this new technology without restrictions. As for the future implementation of AI applications in treatment centers, it is essential that stakeholders (eg, clinicians, researchers, hospital staff) accept and trust the innovative AI-based manufacturing platform. Therefore, the aim of this study was first to identify the factors related to trust in and acceptance of AI technology in medicine and second to assess the relevance of those factors among relevant stakeholders in medicine.

## Methods

### Study Setting

This study is part of the European Union’s (EU) Horizon 2020 project AIDPATH (AI-driven Decentralized Production for Advanced Therapies in the Hospital; grant agreement number 101016909) [22,23]. It is an upcoming state-of-the-art AI application in hospitals, which aims to develop an AI-driven, automated chimeric antigen receptor T cell (CAR-T) manufacturing platform at the point of care as a treatment for acute leukemia and lymphoma. In CAR-T therapy, the patient’s own T cells are removed, genetically modified, and reinfused into the patient in order to find and eliminate tumour cells. Current production is characterized by laborious manual process steps, complex logistics, and a lack of process understanding. This results in long delivery times (up to 21 days) and high costs (approx €320,000, or US $347,890, per treatment) [24,25]. For this reason, AIDPATH is developing a system to fully automate the manufacturing process, from the provision of patient cells to the injection directly in the hospital. An important building block for effective and equitable manufacturing is AI. AI can provide essential process insights into the cell’s characteristics and behavior. This offers a significant benefit for adaptive control of the whole process and the design of personalized process protocols. Furthermore, AI can assist cost-effective platform operation in a smart manufacturing hospital by improving manufacturing schedules and resource management [26]. In general, successful implementation of AIDPATH would serve as an example of an effective AI technology that automates the production and delivery of advanced therapy medicinal products (ATMPs). Furthermore, AI-powered technology can form the basis for a deployable platform for further pilot trials in multiple hospitals and would create a model innovation system for smart manufacturing hospitals [2,22].

In this study, to meet the study objectives, a rapid literature review was conducted, followed by a survey.

### Rapid Literature Review

A rapid literature review of peer- and non-peer-reviewed publications was conducted to identify factors related to trust in and acceptance of AI applications used in medicine. As an alternative method to systematic reviews, a rapid review allows for accelerated synthesis of up-to-date evidence, while efficiently informing latest findings in recent health care research [27]. The peer- and non-peer-reviewed literature needed to be published between 2012 and 2022 in English. Data on attitudes toward AI in relation to prognosis, diagnosis, treatment, and care were included. The search was performed in PubMed/MEDLINE with the following search syntax: ((trust) OR (acceptance) OR (attitude) OR (perspective) OR (perception)) AND ((AI) OR (artificial intelligence) OR (machine learning) OR (deep learning)) AND (((prognosis) OR (diagnosis) OR (treatment) OR (care)) OR ((medic*) OR (clinic*) OR (hospital) OR (smart hospital) OR (health care)) AND ((survey) OR (questionnaire) OR (interview)). The reason for inclusion of only survey-, questionnaire-, or interview-based research in the search terms was due to their direct relevance to our research objectives.

In the non-peer-reviewed literature search, similar terms and time frame of publication were used and the first 10 pages on the Google Search engine were examined to identify other relevant papers and reports by (non)governmental and research organizations. This allowed the study findings to be applicable to a broad range of medical AI applications. Papers were screened, and data were extracted by 2 authors (DS and AA). The selected literature was analyzed to identify key trends and explanatory factors related to trust and acceptance toward medical AI applications. The factors were then grouped into 4 categories: human-related, technology-related, legal and ethical, and additional factors. These factor groups formed the basis of the survey designed to investigate factor relevance. This was performed independently by 2 authors (DS and AA).

### Survey

The survey was reported in accordance with the CHERRIES (Checklist for Reporting Results of Internet E-Surveys) guidelines [28]. The survey in English assessed the relevance of the factors related to trust in and acceptance of novel AI applications in medicine. The survey started with an introduction to AI applications in medicine and AIDPATH, followed by 7 general questions on each participant’s background, including gender, age, the country they worked in, years of experience, the stakeholder group they belonged to, their familiarity with AI applications in medicine, and their general view on AI. In the last question, the following distinction was made between the answer options: “I embrace AI” meant welcoming and using AI as a constituent part of their work or life, “I approve of AI” implied that the participant agreed with the use of AI in their work or life but did not use it themselves, and “I accept AI” referred to acknowledging the use of AI in work or life but not being ready to fully approve it.

In the core section of the survey, the definitions of trust and acceptance were provided as a reference for participants. The core part also consisted of 2 identical lists of 19 factors related to trust and acceptance toward AI applications in medicine. Each factor was categorized into human-related, technology-related, legal and ethical, or additional factors. Human-related factors were linked to AI professionals assessed the relevance of the type of organization the AI professionals were affiliated to and the purpose to innovate with a specific AI application. With respect to health care professionals, the factors were related to the knowledge of AI applications and the attitude toward AI application usage in medicine. In relation to patients, the relevance of the following factors was assessed: general knowledge of AI applications in medicine, the attitude toward AI application usage in medicine, and the patient’s age, gender, and level of education. Furthermore, participants were asked to evaluate the relevance of transparency between all parties involved in AI application use. Technology-related factors related to the performance of AI applications in medicine, the possibility of their integration into existing clinical workflows, a clear balance of risks and benefits of the AI applications, and the explainability and transparency of processes and outcomes. The legal and ethical factors were related to the adequacy of regulations and governance of AI applications in medicine, data use transparency, and clear accountability and responsibility for an AI application. The additional factors were concerned with the environmental sustainability of AI applications and AI’s impact on job availability. For each factor, participants could indicate each factor’s relevance to trust in and acceptance of AI applications from their stakeholder perspective using a Likert scale of 1-5, where 1 stood for “not relevant,” 3 for “not irrelevant, nor relevant,” and 5 for “relevant.” Throughout the survey, “relevant” meant being highly significant for ensuring trust in or acceptance of AI applications, while “irrelevant” meant no significance. The N/A (not applicable) option was available as well for each factor. Open questions at the end of both sections allowed participants to suggest other relevant factors related to trust in or acceptance of AI applications that were not mentioned in the survey. Furthermore, the participants were invited to suggest any other factors, different from trust, deemed important for acceptance of AI applications in medicine.

### Sampling

Using the convenience sampling method [29], AIDPATH Consortium members were requested to invite stakeholders in their network but outside the AIDPATH Consortium to fill in the survey on the SurveyMonkey platform. The survey was distributed by email to members of relevant stakeholder groups to capture their professional perspectives (eg, clinicians, scientists, and policy makers). Data were collected from April to May 2022 and analyzed using Microsoft Excel.

### Data Collection and Analysis

After participants were asked to rate the relevance of each factor from 1 (irrelevant) to 5 (relevant), the mean score of each factor was determined by assigning each response a weight from 1 to 5. Next, means scores were calculated by finding an average of the sum of response values for each question. To visualize the survey responses and compare the mean scores for each factor included in the survey, a spider diagram was charted. This provided an overview of the factors' relevance and their relative importance in influencing both trust and acceptance toward AI applications in medicine. In addition, a scatter plot was created to obtain an overview of the interrelationship between the relevance to trust (x axis) in and acceptance (y axis) of AI applications in medicine. The plot allowed us to identify the degree of relevance of each factor in relation to both trust and acceptance. To classify the factors based on their relevance, score ranges were established. Factors with mean scores from 1 to 3 were considered to be of low relevance, while factors from 4 to 5 were deemed of high relevance. The open-question responses were considered when interpreting numerical data.

### Ethical Considerations

Under Dutch law, no ethical approval was required according to Article 1b of the Dutch Medical Research in Human Subjects Act [30]. However, all participants were informed about the study objectives, their verbal consent was obtained, and all data were processed anonymously. All responses were recorded anonymously. Participants were informed of their right to withdraw from the study at any time without any consequences. They were not financially compensated.

## Results

### Rapid Literature Review

**Figure 1:**
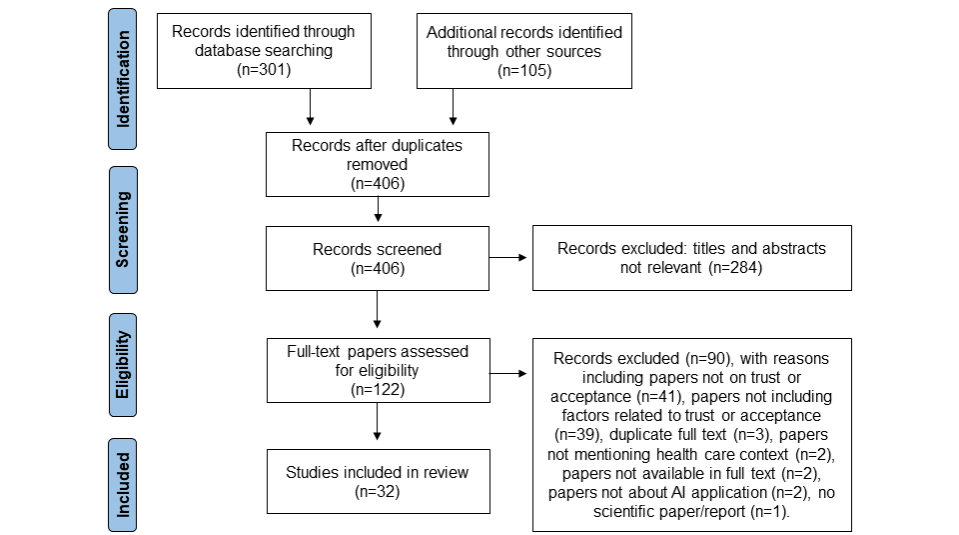
PRISMA flow diagram of the rapid review literature screening. AI: artificial intelligence.

The literature search (Figure 1) yielded 301 hits in the PubMed database and 105 hits through gray literature search and snowballing. After screening titles and abstracts, 284 (70%) records were excluded. After full-text screening, 90 (73.8%) records were excluded primarily due to the absence of concepts of trust or acceptance and a lack of factors related to trust or acceptance in the main text or data-containing figures. As a result, 32 (26.2%) papers and reports [7,9-12,15,16,19,21,31-53] were included in the data analysis.

Overall, the rapid review identified a total of 110 factors related to trust and 77 factors related to acceptance toward medical AI technology. The full list of factors identified through the rapid review with corresponding studies can be found in Multimedia Appendix 1. Tables 1-4 show all factors from the rapid review, each with the frequency of its appearance in the literature and the corresponding overarching umbrella factors. Some factors from a single study are repeated in the same category in Tables 1 and 2 on trust and Tables 3 and 4 on acceptance or within the same category (eg, health care professionals and patients subsections of the human-related factors section). The most frequently reported human-related factors related to trust (Tables 1 and 2) in medical AI applications were knowledge and understanding of AI by health care professionals and knowledge and education of AI among patients. In terms of technology-related factors, accuracy, transparency, reliability, safety, and explainability of medical AI applications and their functioning appeared most often in the literature. Regarding legal and ethical factors, the most frequently occurring factors included fairness and equity of medical AI technology and the privacy and security of personal data handled by the AI systems. The most frequently presented human-related factors related to acceptance (Tables 3 and 4) of medical AI technology were the perceived usefulness and provision of better medical services by the AI technology. Regarding technology-related factors linked to acceptance, performance expectancy, design and output quality, and transparency were stated in the literature most often. A wide range of legal and ethical factors were mentioned in the literature, including adequate regulations of medical AI technology, protection and security of patients’ data, and the allocation of accountability and responsibility for the (mal)functioning of an AI application. There were additional factors related to trust in and acceptance of medical AI technology (Tables 1-4). These included replacement of doctors by machines that lack a human touch and moral support, labor market implications, and environmental sustainability. Three studies also highlighted that acceptance of a medical AI application is directly related to trust in the AI application. Overall, there were fewer factors related to acceptance than those related to trust, whereas most of the overarching umbrella factors were fully represented in both tables. Therefore, an identical list of umbrella factors allocated within the 4 categories (human-related, technology-related, legal and ethical, and additional factors) was used in the survey for investigating the relevance of factors for both trust in and acceptance of AI applications in medicine.

**Table 1:** Human-related factors related to trust (N=110) in medical AI^a^ applications (22/32, 68.8%, studies).

Factor category and factors from the rapid review		Umbrella factors used in the survey
**AI professionals**		
	AI company/provider (n=2, 9.1%)	Type of institution/organization of AI professionals (eg, university, technology company, commercial organization)
	AI role (n=1, 4.5%); perceived helpfulness (n=1, 4.5%)	The purpose to innovate with a specific AI application in medicine (eg, financial vs societal)
**Health care professionals**		
	Knowledge and understanding of AI (n=6, 27.3%); education (n=3, 13.6%)	Knowledge of AI applications in medicine (eg, by means of training and education)
	Expectation of AI (n=1, 4.5%); perceived actionability (ie, clear recommendation for action; n=1, 4.5%); user’s social network (n=1, 4.5%); user’s media consumption (n=1, 4.5%)	Attitude toward AI application usage in medicine (eg, agreeableness, openness, conscientiousness, engagement)
**Patients informed about AI application usage in the hospital**		
	Knowledge/education about AI (n=5, 22.7%); awareness of AI (n=2, 9.1%)	General knowledge of AI applications in medicine
	Openness (to AI health care technologies and to judgments of potential benefits and harms; n=1, 4.5%); perceived benefit and lower concern (n=1, 4.5%); user’s social network (n=1, 4.5%); user’s media consumption (n=1, 4.5%)	Attitude toward AI application usage in medicine (eg, agreeableness, openness, conscientiousness)
	Gender (n=2, 9.1%); age (n=1, 4.5%); type of user (n=1, 4.5%)	Age, gender, level of education
**All parties**		
	Clinicians and patients interaction during AI integration (n=1, 4.5%); human agency and oversight (n=1, 4.5%)	Transparency between all involved parties (AI professionals, health care professionals, patients)

^a^AI: artificial intelligence.

**Table 2:** Other factors related to trust (N=110) in medical AI^a^ applications (22/32, 68.8%, studies).

Factor category and factors from the rapid review		Umbrella factors used in the survey
**Technology-related factors**		
	Accuracy (n=7, 31.8%); reliability (n=5, 22.7%); safety (n=4, 18.2%); design and output quality (n=2, 9.1%); performance expectancy (n=2, 9.1%); ability (n=1, 4.5%); perceived functionality (n=1, 4.5%); self-efficacy (n=1, 4.5%); tool itself (n=1, 4.5%)	Performance of AI applications in medicine (reproducibility of outcomes, accuracy)
	Auditability (n=1, 4.5%); customizability (n=1, 4.5%); understandability (n=1, 4.5%); ease of integration into clinical workflows (n=1, 4.5%); convenience of use (n=1, 4.5%); usability (n=1, 4.5%); (over)alerting and excessive false-positive rate (n=1, 4.5%)	Possibility of integration of AI applications into existing clinical workflows
	Risk and impact mitigation (n=1, 4.5%)	Clear balance of risks and benefits of the AI application
	Transparency (n=6, 27.3%); explainability (n=5, 22.7%); evidence strength (n=2, 9.1%); benevolence (n=2, 9.1%); complexity (n=2, 9.1%); interpretability (n=2, 9.1%); integrity (n=1, 4.5%); predictability (n=1, 4.5%); trialability (n=1, 4.5%); trustworthiness (n=1, 4.5%)	Explainability and transparency of the processes and outcomes
**Legal and ethical factors**		
	Fairness and equity (n=8, 36.4%); adequate regulations, legislation, and governance (n=3, 13.6%); ethical/legal implications (n=1, 4.5%)	Adequacy of the regulations and governance of AI applications in medicine
	Personal data privacy and security (n=8, 36.4%); data used to train AI/cognitive bias (n=2, 9.1%); data sensitivity (n=1, 4.5%); respect and preservation of human dignity (n=1, 4.5%)	Data use transparency
	Accountability (n=3, 13.6%); power-control balance (n=1, 4.5%)	Clear accountability and responsibility of the AI application (machine vs human responsibility)
**Additional factors**		
	Environmental sustainability (n=1, 4.5%)	Environment-friendly AI application
	Replacement of doctor/lack of human touch and moral support when evaluated by AI alone (n=1, 4.5%); labor market implications (n=1, 4.5%)	Impact on job availability (machines replacing humans)

^a^AI: artificial intelligence.

**Table 3:** Human-related factors related to acceptance (N=77) of medical AI^a^ applications (14/32, 43.8%, studies).

Factor category and factors from the rapid review			Umbrella factors used in the survey
**AI professionals**			
	AI company/provider (n=1, 7.1%); brand impact (n=1, 7.1%)	Type of institution/organization of AI professionals (eg, university, technology company, commercial organization)	
	Perceived usefulness (n=3, 21.4%); better medical services/ understanding of disease (n=3, 21.4%); improve the quality of people’s lives (n=2, 14.3%); medical costs (n=2, 14.3%); AI role (eg, saving patients’ time; n=1, 7.1%); miniaturization of hardware (n=1, 7.1%)	Purpose to innovate with a specific AI application in medicine (eg, financial vs societal)	
**Health care professionals**			
	Knowledge and understanding of AI (n=1, 7.1%)	Knowledge of AI applications in medicine (eg, by means of training and education)	
	Behavioral intention to use (n=2, 14.3%); effort expectancy (n=2, 14.3%); perceived ease of use (n=2, 14.3%); perceived usefulness (n=2, 14.3%); intrinsic motivation (n=1, 7.1%); interest in AI (n=1, 7.1%); professional identity (n=1, 7.1%); concerns about benefit to patient care (n=1, 7.1%); general impression of AI (n=1, 7.1%)	Attitude toward AI application usage in medicine (eg, agreeableness, openness, conscientiousness, engagement)	
**Patients informed about AI application usage in the hospital**			
	Knowledge/education about AI (n=1, 7.1%); awareness of AI (n=1, 7.1%)	General knowledge of AI applications in medicine	
	Behavioral intention to use (n=2, 14.3%); general impression (n=1, 7.1%); Interest in topic (n=1, 7.1%)	Attitude toward AI application usage in medicine (eg, agreeableness, openness, conscientiousness)	
	Age (n=1, 7.1%)	Age	
**All parties**			
	Expectations of others (n=2, 14.3%)	Transparency between all involved parties (AI professionals, healthcarehealth care professionals, patients)	

^a^AI: artificial intelligence.

**Table 4:** Other factors related to acceptance (N=77) of medical AI^a^ applications (14/32, 43.8%, studies).

Factor category and factors from the rapid review		Umbrella factors used in the survey
**Technology-related factors**		
	Performance expectancy (n=4, 28.6%); design and output quality (n=4, 28.6%); accuracy (n=2, 14.3%); efficiency (n=1, 7.1%)	Performance of AI applications in medicine (reproducibility of outcomes, accuracy)
	Perceived ease of use (n=2, 14.3%); user-friendliness (n=2, 14.3%); actual system use (n=1, 7.1%); compatibility (n=1, 7.1%); facilitating conditions (n=1, 7.1%)	Possibility of integration of AI applications into existing clinical workflows
	Perceived risk (n=1, 7.1%)	Clear balance of risks and benefits of the AI application
	Transparency (n=3, 21.4%); explainability (n=2, 14.3%); evidence strength (n=1, 7.1%); trustworthiness (n=1, 7.1%)	Explainability and transparency of the processes and outcomes
**Legal and ethical factors**		
	Adequate regulations, legislation and governance (n=2, 14.3%); ethical risks (n=1, 7.1%); political support (n=1, 7.1%)	Adequacy of the regulations and governance of AI applications in medicine
	Data protection/security (n=2, 14.3%); patients’ consent to the continuous collection and processing of data (n=1, 7.1%)	Data use transparency
	Accountability and responsibility (n=2, 14.3%); tort liability (n=1, 7.1%)	Clear accountability and responsibility of the AI application (machine vs human responsibility)
**Additional factors**		
	Replacement of doctor/lack of human touch and moral support when evaluated by AI alone (n=1, 7.1%)	Impact on job availability (machines replacing humans)
	Trust in AI applications (n=3, 21.4%)	Acceptance emerging from trust

^a^AI: artificial intelligence.

### Survey

#### Participants

A total of 22 respondents participated in the survey, of which 18 (82%) completed the questions on trust and 15 (68%) completed the questions on acceptance. No reasons were provided for not completing the survey. Table 5 shows the characteristics of the survey participants, the majority (n=21, 95%) of whom came from European countries, were aged from 40 to 60 years, and had 0-10 or 21-30 years of professional experience.

**Figure 2:**
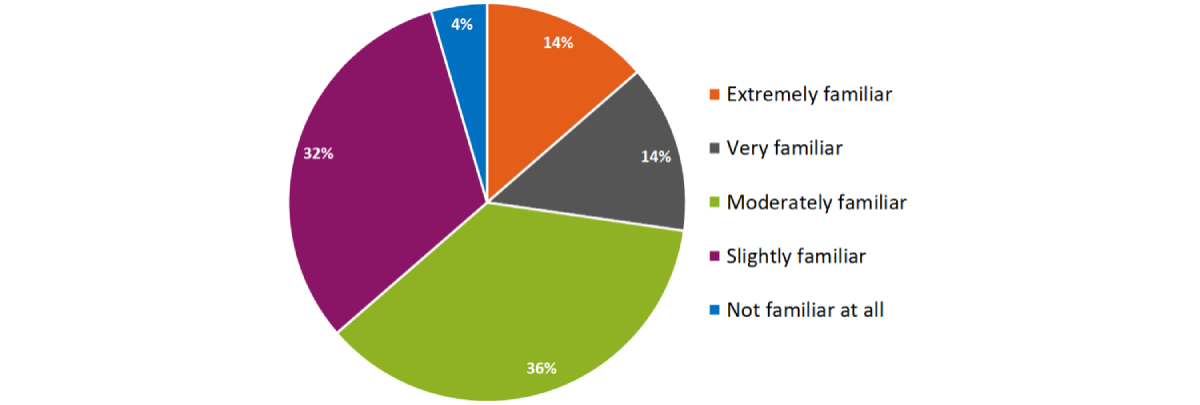
Familiarity with AI-based devices used for clinical purposes (N=22). AI: artificial intelligence.

**Figure 3:**
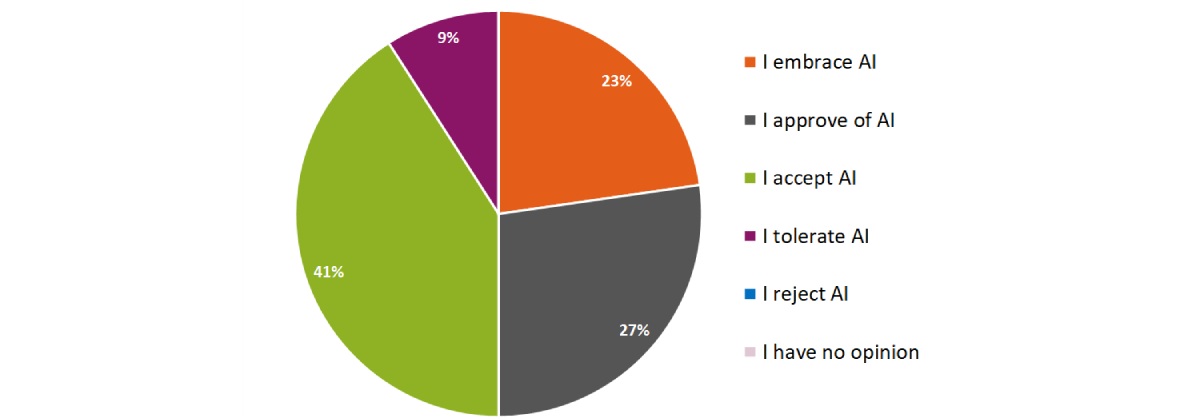
Statement best representing participants’ view when thinking about AI (N=22). AI: artificial intelligence.

Participants were mainly slightly (n=7, 32%) or moderately (n=8, 36%) familiar with AI-based devices used for clinical purposes (Figure 2). In thinking about AI, 9 (41% ) of the participants indicated that the statement “I accept AI” best represents their view, followed by “I approve of AI” (n=6, 27%) and “I embrace AI” (n=5, 23%); see Figure 3.

**Table 5:** Characteristics of the participants (N=22).

Characteristic and type of participant		Participants, n (%)
**Stakeholder group^a^**		
	Researchers	18 (82)
	Technology providers	5 (23)
	Hospital staff	3 (14)
	Policy makers	3 (14)
**Gender**		
	Female	8 (36)
	Male	13 (59)
	Prefer not to say	1 (5)
**Age (years)**		
	≤30	2 (9)
	31-39	3 (14)
	40-49	6 (27)
	50-59	5 (23)
	≥60	6 (27)
**Country of work**		
	Netherlands	11 (50)
	Germany	3 (14)
	Ireland	2 (9)
	Spain	2 (9)
	France	1 (5)
	Hungary	1 (5)
	India	1 (5)
	Italy	1 (5)
**Years of professional experience**		
	0-10	6 (27)
	11-20	4 (18)
	21-30	7 (32)
	31-40	5 (23)

^a^Participants sometimes represented more than 1 stakeholder group.

### Relevance of Factors for Trust in and Acceptance of AI

**Figure 4:**
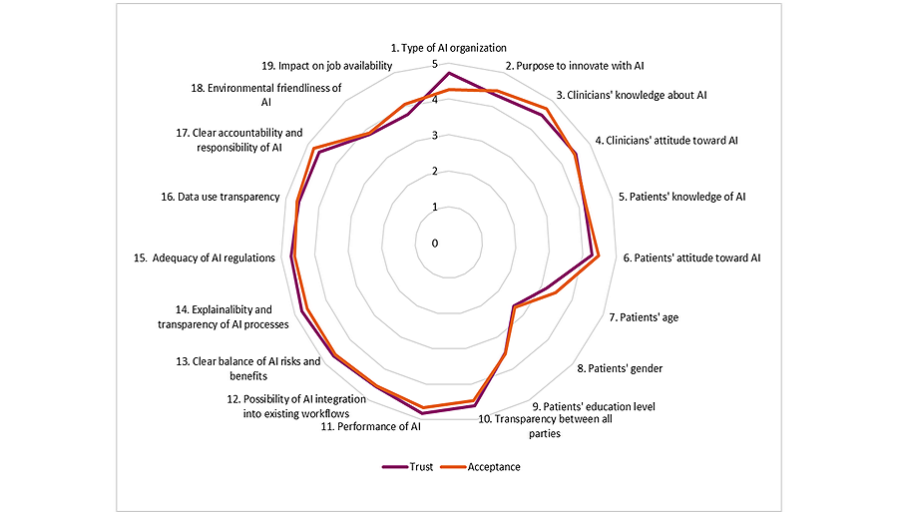
Mean scores of factors’ relevance to trust in and acceptance of AI applications in medicine (N=19). Score=1 means irrelevant; score=3 means not irrelevant, nor relevant; and score=5 means relevant. AI: artificial intelligence.

**Figure 5:**
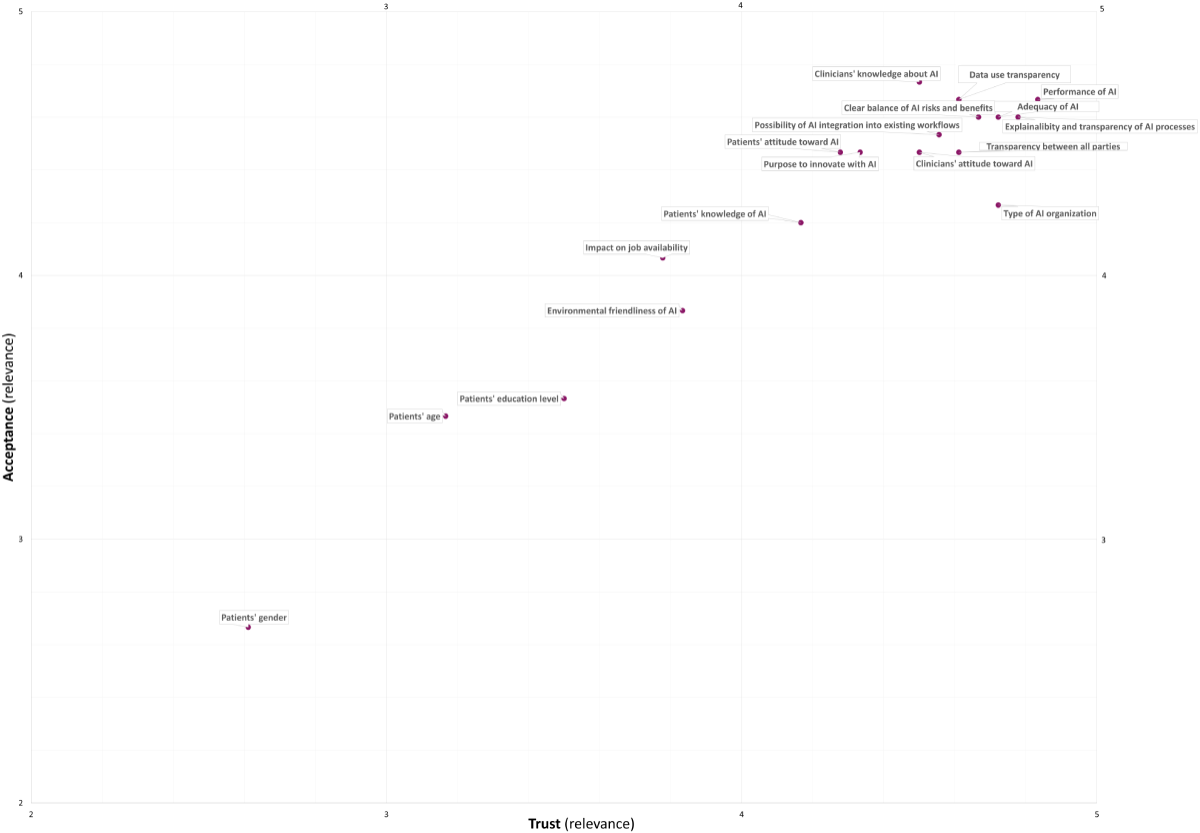
Overview of the relevance of factors related to trust in and acceptance of novel AI applications in medicine (1=not relevant, 5=relevant). AI: artificial intelligence.

In Table 6, the mean scores per factor for its relevance to trust and acceptance are shown. Figure 4 demonstrates a spider diagram with the 19 summarized statements and the corresponding mean scores of relevance to trust in and acceptance of AI applications in medicine. The degrees of relevance of the factors related to trust and to acceptance closely followed each other for all but 1 (5.3%) of the 19 factors. Only the type of AI organization was slightly more relevant to trust than to acceptance toward AI applications in medicine. In Figure 5, a scatter plot displays the combined relevance of the factors related to trust (x axis) and acceptance (y axis) toward medical AI applications. Of the 19 factors included in the survey, 3 (16%) were found to have, on average, low relevance, while the other 16 (84%) had high relevance. There were no factors relevant to acceptance and irrelevant to trust (upper-left section in the plot) and vice versa (bottom-right section in the plot).

**Table 6:** Mean (SD) factor relevance to trust and acceptance (N=22).

Factor	Trust	Acceptance
Type of AI^a^ organization	4.72 (0.75)	4.27 (0.88)
Purpose to innovate with AI	4.33 (0.84)	4.47 (0.64)
Clinicians’ knowledge about AI	4.50 (0.51)	4.73 (0.46)
Clinicians’ attitude towards AI	4.50 (0.51)	4.47 (0.64)
Patients’ knowledge of AI	4.17 (0.62)	4.20 (0.68)
Patients’ attitude toward AI	4.28 (0.57)	4.47 (0.64)
Patients’ age	3.17 (1.04)	3.47 (1.19)
Patients’ gender	2.61 (1.14)	2.67 (1.05)
Patients’ education level	3.50 (0.99)	3.53 (1.19)
Transparency between all parties	4.61 (0.50)	4.47 (0.64)
Performance of AI	4.83 (0.38)	4.67 (0.62)
Possibility of AI integration into existing workflows	4.56 (0.62)	4.53 (0.83)
Clear balance of AI risks and benefits	4.67 (0.49)	4.60 (0.63)
Explainability and transparency of AI processes	4.78 (0.43)	4.60 (0.63)
Adequacy of AI regulations	4.72 (0.57)	4.60 (0.83)
Data use transparency	4.61 (0.50)	4.67 (0.49)
Clear accountability and responsibility of AI	4.61 (0.61)	4.80 (0.41)
Environmental friendliness of AI	3.83 (0.79)	3.87 (0.92)
Impact on job availability	3.78 (1.11)	4.07 (0.88)

^a^AI: artificial intelligence.

#### Factors of Low Relevance

With regard to patients informed about AI application usage in the hospital, participants deemed the patient’s gender, age, and educational level to be of low relevance to trust in and acceptance of novel AI applications in medicine.

#### Factors of High Relevance

The majority of factors were deemed highly relevant to trust in and acceptance of novel AI applications in medicine by participants. Regarding AI professionals, it was observed that the type of institution or organization where AI professionals originated from (eg, university, technology company, commercial organization) and the purpose to innovate with a specific AI application in medicine (eg, financial, societal, or clinical purpose) were considered relevant. Participants reported that the involvement of health care professionals having knowledge of the AI application (eg, by means of training and education) is highly relevant to trust and acceptance. The health care professionals’ attitude toward AI application usage in medicine, comprising their agreeableness, openness, conscientiousness, and engagement, was found to be equally important. Likewise, the patients’ general knowledge of and attitude toward AI application usage in medicine were found to be relevant. The transparency between all involved parties (AI professionals, health care professionals, and patients) was also deemed highly relevant. Technology-related factors were found to be highly relevant, too, in particular the performance of AI applications in medicine (eg, reproducibility and accuracy of outcomes), the possibility of integration of the AI applications into existing clinical workflows, having a clear balance of risks and benefits of the AI applications, and the explainability and transparency of the processes and outcomes. Legal and ethical factors were also considered of high relevance and concerned the adequacy of the regulations and governance of AI applications in medicine, data use transparency, and clear accountability and responsibility of the AI applications (machine vs human responsibility). Additional factors, such as AI applications being environment friendly and the impact of medical AI on job availability (eg, machines replacing human beings), were viewed as factors of high relevance.

#### Other Factors

Participants were able to share other factors that were not mentioned in the survey questions. Factors related to trust included solidarity and understanding the bias and interdomain knowledge of AI in software development, data science, and medicine. Other factors related to acceptance were the extent to which alternatives to AI applications are available, the length of experience, transparency about limitations, reproducibility, risks evaluation, resources, and the fear to use an AI application (ie, fear of making the wrong decision or fear of losing control).

## Discussion

### Principal Findings

This study aimed to identify factors related to trust and acceptance toward medical AI applications by means of a rapid review and to assess their relevance by conducting a survey. Through the rapid review, 19 key factors related to trust in and acceptance of AI-powered medical technologies were identified and subsequently grouped into 4 categories. Our survey results highlight that of all examined factors, 84% (16/19) were considered highly relevant to trust in and acceptance of novel AI applications in medicine. Only the patient’s gender, age, and education level (3/19, 16%) were deemed to be of low relevance by participants.

### Comparison With Prior Work

Previous studies have reported that trust in technology is mainly determined by human characteristics [54], technology-related factors [55], and environment-related factors [56], which is in line with the findings of our survey. According to Tran et al [57], who investigated patients’ perceived benefits and risks of using digital and AI technology in health care, the important factors to consider are the new technologies requiring an overhaul of the current health care system as human care is being replaced by machines and health care professionals becoming sufficiently equipped with increasing knowledge of AI technology. This highlights the importance of several survey factors, including the possibility of AI integration into existing clinical workflows. Therefore, setting features such as understandability, usability, and user-friendliness (factors that frequently appeared in the rapid review) by AI professionals as key goals in the development of novel AI applications would increase the chances of successful integration of AI technology into health care systems. Tran et al [57] also highlighted the increasing importance of data use transparency toward patients and the acute need for clear accountability and responsibility (machine vs human responsibility) concerning the new technology, which also goes hand in hand with the findings from the rapid review and the survey [57]. The patient data handling must be organized in accordance with the existing data protection regulations in respective countries, with additional precautionary measures due to the sensitive nature of such medical data [57]. Shin et al [58] demonstrated that explainability of AI plays a big role in user trust and attitude toward AI. Explainability, along with transparency, was also found to be highly relevant in our study, especially in relation to the AI application processes and outcomes. In addition, Vourgidis et al [59] recommended that AI systems be regularly checked for being up to date, since today’s technology is continuously evolving. This again highlights the relevance of the education of health care professionals, since they are the primary users of medical AI technology and hence need to follow the developments in the field. Yang et al [49] found that gender is not relevant to trust in AI technology in medicine. This agrees with our finding that a patient’s gender has low relevance to trust in and acceptance of AI technology in medicine. Contrary to our findings, it has been reported that younger generations in general have more trust and are more likely to accept AI systems compared to older generations [11]. In our survey, the majority of participants were aged 40-60 years and above and they exhibited a solid awareness of and a positive attitude toward AI technology. Gillespie et al [11] also stated that highly educated people (university level) are more likely to trust and accept AI systems compared to those without a university degree. However, our survey showed that a patient’s educational level has low relevance to trust in and acceptance of medical AI applications.

### Strengths and Limitations

To the best of our knowledge, this is the first study to use a rapid review of the latest literature to identify factors related to trust in and acceptance of AI applications in medicine in order to create a survey to evaluate their relevance and the attitudes of health care stakeholders toward implementation of medical AI applications. However, the study has several limitations. Since a large number of papers and reports in the rapid review did not provide sufficient context for the factors for trust or acceptance, there could have been an increased risk of personal bias during interpretation and categorization of those factors. Furthermore, some studies did not clarify whether the reported factors were related to only trust or only acceptance, which could also lead to possible misinterpretation. To minimize the effect of such bias and misinterpretation, a third reviewer (author HJMV) was consulted in such cases. Another limitation is the relatively small number of papers included in the rapid review, given the breadth of the topic. However, this rapid review was intentionally conducted focusing on the most relevant and recent literature to provide an initial overview and highlight key themes in a time-efficient manner. We aimed to provide a starting point that formed the basis for the survey. In addition, the number of participants included in the survey can be considered relatively low, which was caused by difficulties in recruiting participants and the time-constrained nature of the study. However, sufficient diversity in participant characteristics (ie, gender, age, country of work, and years of professional experience) was achieved, which could be considered more important in terms of validity of the study findings. Even though the survey benefited from a sample with a wide diversity in participant characteristics, one of the limitations to consider is the underrepresentation of certain stakeholder groups, in particular technology providers, policy makers, and hospital staff members other than clinicians. If these groups had been included in the survey, different patterns in factor relevance might have been observed, potentially shedding light on additional concerns or challenges associated with AI applications in medicine. Moreover, when considering the relevance of factors assessed through the survey, which were predominantly highlighted by researchers, it is important to note that these factors might be readily attainable or already well established within this specific stakeholder group. As a result, these factors may not necessarily represent challenges or barriers for this particular group, as they are already well versed in the aspects related to trust and acceptance.

### Recommendations for Future Research

The results of this study can be valuable for various stakeholders involved in the implementation of novel AI applications, since trust and acceptance building remains a focus point throughout the different stages, including the pilot, implementation, evaluation, and monitoring phases of the process. In the survey, participants shared other factors related to trust in and acceptance of AI applications in medicine that were not included in the survey. However, due to a lack of context, it is not entirely clear what was meant by some of these factors; since these are open to interpretation, follow-up research is required to better understand this. In addition, further research is needed to gain insight into the reasons participants considered factors to be of low or high relevance. Regarding the currently underrepresented stakeholder groups in the survey, more research is required to gain insight into the perspectives of policy regulators, technology providers, and hospital staff members. Next, once the implementation of a novel AI technology, such as the AIDPATH system, becomes clear from the trust and acceptance point of view, it would be beneficial to conduct a workshop with experts from the AI and biotechnology fields to identify technical challenges of implementation. This is crucial since, according to the survey results, the technical robustness and clarity of AI applications is a prerequisite for trust and acceptance exhibited toward this technology by stakeholders.

### Recommendations for Implementation

By considering the factors that are most relevant in the AI technology adoption process, the implementers can facilitate trust in and acceptance of medical AI applications among their users and other stakeholders. Furthermore, the knowledge of the factors with high relevance to stakeholders can predict concerns the potential users might have regarding the new AI technology and act upon these concerns to implement the AI application efficiently and in a timely manner. There are several ways in how the results of the survey could be used by AI implementers, such as smart hospitals, to build trust and acceptance among various stakeholder groups. For instance, the highly relevant factor of knowledge and understanding of AI among health care professionals could be addressed by providing information about medical AI to clinicians in the form of conferences and educational workshops. These initiatives can ensure that health care professionals remain updated on significant changes in AI technology, facilitating its accurate utilization. Similarly, patients could be informed of medical AI technology through patient information initiatives in (smart) hospitals and within patient communities. The highly relevant technology-related factors could be used by technology developers and scientific researchers as guidance in the development of novel AI technology. For regulators and policy makers, it is crucial to know that users and other stakeholders consider data use transparency and fairness and equity to be of utmost importance regarding novel medical AI technology. Indeed, data privacy is a crucial and ever-so-present topic in legislation and regulations, but it needs to be constantly reviewed by policy makers due to the newness of AI in health care and the speed of its development. The legal aspects of software containing AI have been subjected to the Medical Device Regulation (MDR) [60]. For the acceptance of AI, its implementation in MDR-compliant solutions is invaluable. The tasks of policy makers could involve the risk assessment of various data breaches related to AI in medicine with continuous updating of regulations related to data security and privacy within the field of medical AI. Furthermore, both policy makers and AI professionals have to ensure the maintenance of fairness and equity of AI technology usage.

### Conclusion

This study identified and assessed the relevance of factors for trust in and acceptance of AI applications in medicine. The survey demonstrated that the majority of the identified human-related, technology-related, and legal and ethical factors for trust in and acceptance of novel AI applications in medicine were considered by stakeholders to be of high relevance. Taken together, these findings and subsequent recommendations could be used by any implementers of medical AI, such as (smart) hospitals, AI technology organizations, biotechnology research institutes, and policy makers, to facilitate smooth and timely adoption of novel AI applications in medicine.

## Appendix

Multimedia Appendix 1 Identified factors and corresponding studies included in the rapid review.

## Abbreviations

AI: artificial intelligence
AIDPATH: AI-powered Decentralized Production for Advanced Therapies in the Hospital
CAR-T: chimeric antigen receptor T cell
MDR: Medical Device Regulation

## Data Availability

The data used and analyzed in this study are available from the corresponding author upon reasonable request.
