# Supplementary material for: Trust in and Acceptance of Artificial Intelligence Applications in Medicine: Mixed Methods Study
Source: JMIR Hum Factors. 2024 Jan 17;11:e47031. doi: 10.2196/47031 (PMC10831593; doi:10.2196/47031)
Supplement: Multimedia Appendix 1 [file humanfactors_v11i1e47031_app1.doc]

**Multimedia Appendix 1** **- Identified factors and corresponding studies included in the rapid review**

1. **Factors related to trust**

| **Factor categories** | **Factors from the rapid review** | **Study (author, year)** |
| --- | --- | --- |
| **Human-related factors**  (AI professionals,  healthcare  professionals,  patients) | *AI professionals:* |  |
|  | AI company/provider (n=2) | Alaqra et al. (2021)  Jermutus et al. (2022) |
|  | AI role (n=1)  Perceived helpfulness (n=1) | Asan et al. (2020)  Hui et al. (2021) |
|  | *Healthcare professionals:* |  |
|  | Knowledge and understanding of AI (n=6)  Education (n=3) | Alaqra et al. (2021)  Asan et al. (2020)  Brady (2022)  Diprose et al. (2020)  Jermutus et al. (2022)  Jobin et al. (2019)  Schwartz et al. (2022)  Tucci et al. (2021)  Yang et al. (2019) |
|  | Expectation of AI (n=1)  Perceived actionability (i.e. clear recommendation for action) (n=1)  User’s social network (n=1)  User’s media consumption (n=1) | Jermutus et al. (2022)  Schwartz et al. (2022) |
|  | *Patients informed about AI application usage in the hospital:* |  |
|  | Knowledge/education about AI (n=5)  Awareness of AI (n=2) | Asan et al. (2020)  Brady (2022)  Gillespie et al. (2021)  Jobin et al. (2019)  Tucci et al. (2021)  Yang et al. (2019) |
|  | Openness (to AI healthcare technologies and to judgments of potential benefits and harms) (n=1)  Perceived benefit and lower concern (n=1)  User’s social network (n=1)  User’s media consumption (n=1) | Antes et al. (2021)  Jermutus et al. (2022) |
|  | Gender (n=2)  Age (n=1)  Type of user (n=1) | Jermutus et al. (2022)  Yang et al. (2019) |
|  | *All parties:* |  |
|  | Clinicians and patients interaction during AI integration (n=1)  Human agency and oversight (n=1) | Yap et al. (2022)  Gillespie et al. (2021) |
| **Technology-related factors** | Accuracy (n=7)  Reliability (n=5)  Safety (n=4)  Design and output quality (n=2)  Performance expectancy (n=2)  Ability (n=1)  Perceived functionality (n=1)  Self-efficacy (n=1)  Tool itself (n=1) | Alaqra et al. (2021)  Gillespie et al. (2021)  Hui et al. (2021)  Jermutus et al. (2022)  Jobin et al. (2019)  Matheny et al. (2022)  Rajpurkar et al. (2022)  Schwartz et al. (2022)  Toreini et al. (2019)  Tucci et al. (2021)  Williams et al. (2021)  Yang et al. (2019)  Yap et al. (2022)  Zhang et al. (2021a)  Zhang et al. (2021b) |
|  | Auditability (n=1)  Customisability (n=1)  Understandability (n=1)  Ease of integration into clinical workflows (n=1)  Convenience of use (n=1)  Usability (n=1)  (Over-)alerting and excessive false positives rate (n=1) | Allen (2021)  Jermutus et al. (2022)  Joshi et al. (2022)  Rajpurkar et al. (2022)  Toreini et al. (2019) |
|  | Risk and impact mitigation (n=1) | Gillespie et al. (2021) |
|  | Transparency (n=6)  Explainability (n=5)  Evidence strength (n=2)  Benevolence (n=2)  Complexity (n=2)  Interpretability (n=2)  Integrity (n=1)  Predictability (n=1)  Trialability (n=1)  Trustworthiness (n=1) | Asan et al. (2020)  Diprose et al. (2020)  Jermutus et al. (2022)  Jobin et al. (2019)  Matheny et al. (2022)  Rajpurkar et al. (2022)  Schwartz et al. (2022)  Toreini et al. (2019)  Tucci et al. (2021)  Williams et al. (2021)  Zhang et al. (2021a) |
| **Legal and ethical factors** | Fairness and equity (n=8)  Adequate regulations, legislation and governance (n=3)  Ethical/legal implications (n=1) | Allen (2021)  Gillespie et al. (2021)  Jobin et al. (2019)  Matheny et al. (2022)  Schwartz et al. (2022)  Toreini et al. (2019)  Tucci et al. (2021)  Williams et al. (2021) |
|  | Personal data privacy and security (n=8)  Data used to train AI /cognitive bias (n=2)  Data sensitivity (n=1)  Respect and preservation of human dignity (n=1) | Alaqra et al. (2021)  Allen (2021)  Asan et al. (2020)  Gillespie et al. (2021)  Gillie et al. (2020)  Hui et al. (2021)  Jermutus et al. (2022)  Jobin et al. (2019)  Williams et al. (2021)  Yap et al. (2022) |
|  | Accountability (n=3)  Power-control balance (n=1) | Gillespie et al. (2021)  Jermutus et al. (2022)  Jobin et al. (2019)  Williams et al. (2021) |
| **Other factors** | Environmental sustainability (n=1) | Jobin et al. (2019) |
|  | Replacement of doctor/lack of human touch and moral support when evaluated by AI alone (n=1)  Labour market implications (n=1) | Jobin et al. (2019)  Yap et al. (2022) |

1. **Factors related to acceptance**

| **Factor categories** | **Factors from the rapid review** | **Study (author, year)** |
| --- | --- | --- |
| **Human-related factors**  (AI professionals,  healthcare  professionals,  patients) | *AI professionals:* |  |
|  | AI company/provider (n=1)  Brand impact (n=1) | Joshi et al. (2022)  Tamori et al. (2022) |
|  | Perceived usefulness (n=3)  Better medical services/understanding of disease (n=3)  Improve the quality of people’s lives (n=2)  Medical costs (n=2)  AI role (e.g., saving patients’ time) (n=1)  Miniaturisation of hardware (n=1) | Jauk et al. (2021)  Kovalchuk et al. (2022)  Shah et al. (2022)  Tamori et al. (2022)  Walter et al. (2020)  Zheng et al. (2021) |
|  | *Healthcare professionals:* |  |
|  | Knowledge and understanding of AI (n=1) | Tamori et al. (2022) |
|  | Behavioural intention to use (n=2)  Effort expectancy (n=2)  Perceived ease of use (n=2)  Perceived usefulness (n=2)  Intrinsic motivation (n=1)  Interest in AI (n=1)  Professional identity (n=1)  Concerns about benefit to patient care (n=1)  General impression of AI (n=1) | Cornelissen et al. (2022)  Jauk et al. (2021)  Joshi et al. (2022)  Kovalchuk et al. (2022)  Tamori et al. (2022  Van Bussel et al. (2022) |
|  | *Patients informed about AI application usage in the hospital:* |  |
|  | Knowledge/education about AI (n=1)  Awareness of AI (n=1) | Tamori et al. (2022)  Shah et al. (2022) |
|  | Behavioural intention to use (n=2)  General impression (n=1)  Interest in topic (n=1) | Kovalchuk et al. (2022)  Tamori et al. (2022) |
|  | Age (n=1) | Shah et al. (2022) |
|  | *All parties:* |  |
|  | Expectations of others (n=2) | Tamori et al. (2022) |
| **Technology-related factors** | Performance expectancy (n=4)  Design and output quality (n=4)  Accuracy (n=2)  Efficiency (n=1) | Cornelissen et al. (2022)  Fujumori et al. (2022)  Jauk et al. (2021)  Matheny et al. (2022)  Tamori et al. (2022)  van Bussel et al. (2022)  Zhang et al. (2021a)  Zheng et al. (2021) |
|  | Perceived ease of use (n=2)  User-friendliness (n=2)  Actual system use (n=1)  Compatibility (n=1)  Facilitating conditions (n=1) | Cornelissen et al. (2022)  Fujumori et al. (2022)  Jauk et al. (2021)  Kovalchuk et al. (2022)  Tamori et al. (2022)  Walter et al. (2020) |
|  | Perceived risk (n=1) | Matheny et al. (2022) |
|  | Transparency (n=3)  Explainability (n=2)  Evidence strength (n=1)  Trustworthiness (n=1) | Fujumori et al. (2022)  Haggenmüller et al. (2021)  Matheny et al. (2022)  Zhang et al. (2021a) |
| **Legal and ethical factors** | Adequate regulations, legislation and governance (n=2)  Ethical risks (n=1)  Political support (n=1) | Matheny et al. (2022)  Tamori et al. (2022)  Zheng et al. (2021) |
|  | Data protection/security (n=2)  Patients’ consent to the continuous collection and processing of data (n=1) | Tamori et al. (2022)  Walter et al. (2020) |
|  | Accountability and responsibility (n=2)  Tort liability (n=1) | Matheny et al. (2022)  Tamori et al. (2022)  Zheng et al. (2021) |
| **Other factors** | Replacement of doctor/lack of human touch and moral support when evaluated by AI alone (n=1) | Zheng et al. (2021) |
|  | Trust in AI application (n=3) | Cornelissen et al. (2022)  Gillespie et al. (2021)  Joshi et al. (2022) |
